# Trials within trials? Researcher, funder and ethical perspectives on the practicality and acceptability of nesting trials of recruitment methods in existing primary care trials

**DOI:** 10.1186/1471-2288-10-38

**Published:** 2010-04-30

**Authors:** Jonathan Graffy, Peter Bower, Elaine Ward, Paul Wallace, Brendan Delaney, Ann-Louise Kinmonth, David Collier, Julia Miller

**Affiliations:** 1 NIHR School for Primary Care Research, University of Cambridge, Cambridge, UK; 2 NIHR School for Primary Care Research, University of Manchester, Manchester, UK; 3 NIHR CRNCC Primary Care Research Network, London, UK; 4 Department of General Practice and Primary Care, King's College London, London, UK; 5 William Harvey Research Institute, Queen Mary University of London, London, UK; 6 NIHR CLAHRC for Greater Manchester, School of Medicine, Manchester, UK

## Abstract

**Background:**

Trials frequently encounter difficulties in recruitment, but evidence on effective recruitment methods in primary care is sparse. A robust test of recruitment methods involves comparing alternative methods using a randomized trial, 'nested' in an ongoing 'host' trial. There are potential scientific, logistical and ethical obstacles to such studies.

**Methods:**

Telephone interviews were undertaken with four groups of stakeholders (funders, principal investigators, trial managers and ethics committee chairs) to explore their views on the practicality and acceptability of undertaking nested trials of recruitment methods. These semi-structured interviews were transcribed and analysed thematically.

**Results:**

Twenty people were interviewed. Respondents were familiar with recruitment difficulties in primary care and recognised the case for 'nested' studies to build an evidence base on effective recruitment strategies. However, enthusiasm for this global aim was tempered by the challenges of implementation. Challenges for host studies included increasing complexity and management burden; compatibility between the host and nested study; and the impact of the nested study on trial design and relationships with collaborators. For nested recruitment studies, there were concerns that host study investigators might have strong preferences, limiting the nested study investigators' control over their research, and also concerns about sample size which might limit statistical power. Nested studies needed to be compatible with the main trial and should be planned from the outset. Good communication and adequate resources were seen as important.

**Conclusions:**

Although research on recruitment was welcomed in principle, the issue of which study had control of key decisions emerged as critical. To address this concern, it appeared important to align the interests of both host and nested studies and to reduce the burden of hosting a recruitment trial. These findings should prove useful in devising a programme of research involving nested studies of recruitment interventions.

## Background

Recruitment to studies such as randomized trials is traditionally seen as highly problematic [1], and trials in primary care are no exception [2]. Indeed, primary care researchers encounter particular difficulties because of the need to engage clinicians working in dispersed settings, where patients attend intermittently and where there is limited time and multiple competing priorities. Even experienced researchers are often uncertain how best to motivate primary care patients and professionals to participate [3]. Recent years have seen advances in our understanding of the recruitment process [4], the barriers that exist [5], and the psychological and social mechanisms which are involved in the decision to participate in a trial and contribute to research [6-8]. Despite these developments, rigorous evidence concerning the best ways of improving recruitment in primary care is sparse. The Cochrane CENTRAL database of controlled trials includes many thousands of records, but recent systematic reviews of trials of methods of improving recruitment to trials found only limited numbers of relevant studies [9,10], while reviews of the role of incentives are equally limited [11]. Much of the literature in this field reports single 'case studies' investigating whether a particular method or approach succeeded in the context of a given trial [12-15]. Factors that are identified as key to success in trials which have recruited satisfactorily are often also present in those that have not recruited [16] which casts doubt on their significance. Recruiting for science is not underpinned by a science of recruitment.

Like any intervention, a rigorous test of the effectiveness of a recruitment method is a randomized trial comparing one recruitment method with an alternative, conducted in the context of an ongoing 'host' trial. For example, Donovan et al randomized men with localized prostate cancer to see a nurse or surgeon for an 'information appointment' in which they were asked to consent to a trial comparing surgery, radiotherapy, and active monitoring. The results showed that there was a small reduction (4%) in recruitment rate in the group seeing a nurse, but that the costs of using surgeons were higher [17].

Although some 'nested' trials have been successful, the relatively small number identified by the published systematic reviews [9,10] suggests that they are not unproblematic. Indeed, there are a host of potential scientific, logistical and ethical obstacles. For example, there may be too few units of analysis available for randomization to achieve reasonable power. It may be difficult to get investigators to agree to randomize to different methods, given that recruitment is such a major issue and a consistent cause of delay. There may be ethical concerns if different patient populations are approached in different ways. Funders may have concerns about the impact of the nested study on the progress of the host trial. One of the problems with interpreting the results of single trials of nested recruitment interventions is that their effect may be influenced by the context in which the study is done, and the results of a single nested study may not generalize.

Clearly, undertaking nested trials of recruitment methods presents a major challenge, but the potential rewards would be high if nested trials contributed to the evidence base and eased the difficulties in recruitment that most investigators experience [1,2]. However, at present nested studies are largely designed and delivered in an ad hoc way in the context of individual trials, limiting their impact. Developing a reliable and rigorous evidence base may require a more systematic approach to conducting nested trials, involving investigators testing recruitment interventions across a number of host trials simultaneously to maximize sample size and generalizability. Although a number of potential barriers to nested recruitment studies are described above, it is not known if this is comprehensive, and there is no indication of the relative importance of the different issues. Therefore, we conducted a study involving key stakeholders working on the design, delivery and monitoring of primary care trials. The aim of the study was to explore the perspectives of stakeholders concerning the acceptability and practicality of nesting trials of recruitment methods in existing trials.

## Methods

We used a qualitative research design, in line with the Medical Research Council framework for the development of trials to evaluate complex interventions [18,19], because we wanted to explore the scientific, logistical and ethical considerations that might be relevant to nested recruitment trials in primary care. We adopted a semi-structured interview format so that participants could consider and explain their perspectives on the proposed approach, but opted to conduct these by telephone to reduce travel and maximize convenience for participants, several of whom held senior roles.

Although patients and collaborating clinicians have important roles in clinical trials, we opted to focus primarily on those involved at an early phase in decisions about trial design. The key stakeholders were identified as principal investigators and trial managers, along with representatives of organisations funding trials and people serving as ethics committee chairs. We sought to interview around 20 stakeholders from these groups because we believed that doing so would enable us to include a reasonable range of perspectives.

### Sampling strategy

To identify principal investigators and trial managers we purposively sampled the National Institute of Health Research Primary Care Research Network (NIHR PCRN) portfolio database, which includes all trials receiving NHS Service Support. We identified current trials and classified these according to design (i.e. cluster versus individual), population (routine primary care populations versus 'special' groups such as adolescents) and intervention type (clinical versus health services research). We then selected potential informants at random from each group, randomly selecting an alternative if they declined.

The remainder of our sample was sought from funders and ethics committees. The major UK public research funding bodies (Medical Research Council, NIHR Health Technology Assessment Programme, NIHR Evaluation, Trials and Studies Coordinating Centre and NIHR Central Commissioning Facility) were approached to identify a senior manager. We contacted the National Research Ethics Service to identify ethics committee chairs and independently approached two others who were known to members of the team from previous research work.

Ethical approval was given by the University of Manchester Senate Ethics Committee. Potential respondents were approached via email and telephone and invited to take part in a telephone interview. They were asked to consent to their interviews being audio-recorded.

### Interview schedule

Semi-structured interview schedules were devised, with minor variations to make them relevant to each stakeholder group. The schedules were piloted with senior researchers and modified during the project in the light of experience in the initial interviews. A document explaining the proposal (i.e. to work with multiple research teams to test alternative recruitment approaches across multiple trials) was emailed to participants prior to interview. The document also outlined potential recruitment interventions, which varied in size, complexity, type and the resources they might require (Table 1). Interviews were conducted by EW. They began with general questions about primary care research experience and explored views about recruitment problems (Table 2). Respondents were then asked to refer to the document outlining potential recruitment interventions and were questioned about potential scientific, logistical and ethical issues.

**Table 1:** Summary of potential recruitment interventions provided to participants before interview

	Unit of Allocation^1^	Recruitment Stage^2^	Type of Intervention
Financial incentives to patients and professionals (e.g. payment for recruitment, lottery etc)	Trial, cluster, individual	1, 2, 3, 4	Incentives

Attachment of additional, dedicated research nurses for sessions in participating centres	Trial	2, 3, 4	Resources

Showing a DVD of previous trial participants discussing their experiences of being involved in research.	Cluster, individual	1, 2, 3	Attitudes of patients and professionals

Mass media approaches to change attitudes to trials among patients	Trial	3, 4	Attitudes of patients

Educational incentives to clinicians. e.g. seminar on trials and research methods	Trial, cluster	1, 2	Attitudes of professionals

Training for clinicians in seeking consent for trials	Trial, cluster	2	Attitudes of professionals

Option to refer patients to a dedicated research centre or hub	Trial, cluster	2	Incentives for professionals

Support for investigators on project management and monitoring approaches, with in-built contingency planning	Trial	Trial planning	Advice and support for trial recruitment

^1 ^Unit of allocation for the recruitment intervention: (Trial, Cluster within a trial, Individual patient)

^2 ^Recruitment stage that intervention is designed to improve:

Trial planning and organization;

Stage 1: Professional consent to participate in trial;

Stage 2: Professional recruitment of patients;

Stage 3: Patient consent to participate in trial;

Stage 4: Retention of patient in trial

**Table 2:** Outline of interview schedule

Past experiences of primary care studies
How decisions on recruitment strategies are made
Barriers to recruitment and potential solutions
Views about feasibility of nested recruitment methods interventions:
- Advantages of nesting recruitment interventions
- Disadvantages of nesting recruitment interventions
- Risks to main study of nesting recruitment interventions
- Would it matter what sort of main trial was being conducted?
- Would the timing of nested studies matter?
- Discussion of recruitment interventions circulated in advance (*Table 1*)

*Topics discussed with subgroups:*
- Personal views if asked to nest a recruitment study *(PIs)*
- Ethical implications *(Ethics)*
- Would funding body support/incentivize nested studies? *(Funders)*

The interviews were recorded using teleconferencing facilities, downloaded and sent for 'intelligent' transcription (i.e. excluding hesitations and non-verbal expressions). The transcriptions were analyzed thematically using a pre-determined framework which was derived from the interview schedule but then adapted and revised during the analysis [20]. Each transcript was read and annotated by EW and one other member of the team. Following this, a summary of the data was prepared, and categorized according to the revised framework. This was discussed at a meeting of the research team and key themes were identified. During the course of the analysis, the researchers compared their interpretations and also contrasted the perspectives of the different groups of participants.

## Results

Of 24 people approached, 20 were interviewed: 7 principal investigators, 6 trial managers, 4 representatives of funding bodies and 4 ethics committee representatives (3 committee chairs and 1 regional committee officer). One individual was both a representative of a funding body and a principal investigator. Three respondents were previously known to the interviewer. Three declined to participate (2 principal investigators and one ethics committee chair) and one national funding body did not consider their work relevant to our enquiries.

All informants were knowledgeable about primary care research recruitment difficulties. Most trial managers and principal investigators had considerable experience of research recruitment, usually in primary care settings. Although the experience of ethics committee and funding body representatives varied more widely, all had some primary care experience.

### The benefits of nested recruitment interventions

Interviewees were generally receptive to the idea of researching recruitment interventions and agreed that researchers often struggled to achieve recruitment targets. They felt such research could challenge preconceptions and add to the evidence base on recruitment. As a result, researchers would be able to target resources where they might be most effective.

'Because you need to find out whether these things work, and the only way of finding out if they work is by trialing it, or doing a randomized trial .... It would be really useful to know the answer to these questions, whether this actually works.' (Respondent 010, Trial Manager, TM)

Whilst the knowledge generated through nested recruitment studies would primarily benefit the wider research community, interviewees also identified more immediate benefits which might accrue to the host study. These included access to new resources and training, as well as the potential to improve recruitment.

'As a PI, what you'd be thinking about is, was this a little bit more resource?... If this was an extra bit of resource then that would be a huge advantage. The other advantage would be as with all research ... that you might actually get an answer to the question because it's a difficult thing to do and we know it's difficult; if we thought we were contributing to finding out ways of making it better, then I think we'd be delighted.' (017, Principal Investigator, PI)

Several commented that researching recruitment by conducting nested studies was likely to be cost effective. The process of conducting such studies would also prompt the exchange of ideas and collaboration between research teams.

'You'd maybe get experience of something you hadn't thought of, an approach that is different from what you would have thought of putting into the protocol to begin with.... Advice and involvement with other triallists and people that might help.' (011, TM)

### Issues for host studies

Respondents differentiated between potential disadvantages for host studies and those for the nested trial. Concerns for host studies centred on management burden, trial complexity, compatibility between the two studies, and impacts on trial design and relationships with collaborators. There were also concerns about the impact on patients if the consent process was made more complex.

#### Organizational burden

Interviewees expressed concerns about study management burden, given that managing the host trial was already challenging. Some voiced concerns about an *additive *effect of nested trials and whether additional procedures would impact on current workload.

'My immediate thought is, it's just one more thing to remember and to try and keep on top of. I don't know if you've run a clinical trial, but just keeping a track of where you're up to with everybody is a nightmare.' (005, PI)

'Some studies already struggle with being quite complex; fitting something else in depends on how much it impinges on that study.' (012, PI)

The presence of the additional intervention could also *interact *with the host study to compound complexity and burden.

'Well they [nested studies] just complicate things, don't they? They complicate things organizationally; they could complicate things in the sense that if the nested study goes wrong, what happens to the main study? What are the implications for the main study? If there's a major adverse event in the nested study, what does that mean for the host study?' (003, PI)

#### Methodological compatibility

The impact of a nested study would also depend on compatibility - the degree to which both studies used similar procedures and criteria.

'I don't really see any [problems] provided it didn't compromise the design of the original trial set-up. So provided it could actually be nested within and wasn't really pushing the boundaries of the inclusion or exclusion criteria, or the length of recruitment, provided then it wasn't going to scupper the trial it's nested in ... As long as the nested design fitted initially with the particular trial I think it would be fine.' (004, TM)

Several respondents were concerned about impacts on the host trial design. Common quality safeguards applied to trials should also be applied to nested trials.

'There's the whole question of blinding and outcome assessment and all the rest of it. It's a separate study so it's a question of making sure that the people delivering the different interventions are separate from the outcome assessors and so on.' (001, Funder)

In certain contexts, nesting could subvert the design of the host trial, by selectively increasing recruitment in one arm of a trial, or modifying the type of participants recruited.

'If you have a cluster trial and your cluster size is not very big, I suppose there is a danger that by doing your.... well there is a chance that by intervening in your clusters, you affect recruitment differentially to different groups, because if the clusters are in effect the randomized unit and they're not well balanced across the trial intervention, the additional trial interventions that you're performing ...might actually unbalance randomization.' (015, PI)

The particular recruitment interventions discussed are considered below, but as a general point it was noted that certain interventions could even reduce recruitment.

'It might be that some of these interventions actually are not just helpful, but are harmful....I might say about [travelling to] the dedicated hub: if patients don't want to do that, it could actually in some places have an adverse effect on recruitment.' (015, PI)

#### Relationships with collaborators

If the nested study involved allocating different levels of resources for recruitment, this could affect relationships between the study team and clinical collaborators. Also collaborators who had strong preferences for one recruitment approach might not agree to random allocation.

'Well the first thing that springs to mind is if you get your GPs together for a meeting and discuss that, then I could see some going, "Oh, but I only wanted to have that bit of the study", and - because you even get it sometimes with double blinds and stuff where they're going, "But I want that arm of the study," and you say, "Well, no you can't, we're randomizing it." I could just imagine potentially there would be rather more of that: "Well how come they get to see the DVDs and we have to deal with this bit?".' (002, TM)

'Money is always a difficult thing. Because if you're paying people - then you potentially may randomize them to the arm that doesn't pay them - some people may be less than happy.' (019 PI/Funder)

#### Impact on participants

Unsurprisingly, the ethics committee chairs had concerns that the additional complexity of a nested study might increase the burden on patients, and that this might impact differently on different patient populations.

'One would need to look again at the health profiles and social profiles of the participants. So if they're quite frail, if they've got serious mental health problems, is this putting an unreasonable burden on them? Does the outcome overcome the burden or balance against the burden.' (007, Ethics)

'So I would be perfectly happy for you to go and do it in different ways, but this needs to be thought about: what is the framework within which this is being done, so that you can ensure that no patient is demonstrably worse off as a result of participating in it?' (009, Ethics)

### Issues for nested recruitment studies

#### Control of the research

A recurring theme was the concern that the nested study should not jeopardize the host trial. As a result, although research on recruitment was seen as useful, this support was conditional and depended on the particular circumstances. If host study investigators or clinical collaborators had strong preferences for one recruitment approach, this might conflict with the intention of the nested study to allocate different patients or sites to different conditions. Or, if overall recruitment proved inadequate as the study progressed then the priorities of the host study could override those of the nested study. This might involve stopping an intervention in one arm, or shifting additional resources to the poorly performing arm.

'The trouble is, if your trial is struggling... you need to do anything you can, so you would then potentially swamp the effect of any intervention.' (015, PI)

#### Study design

Participants made a number of comments about the design of nested methods trials. Essentially, all aspects of study design needed to be addressed in order to produce a coherent research plan. For example, it was important to achieve adequate statistical power in the nested study.

'The difficulties are that if recruitment is your outcome then you actually need quite large numbers to see differences between different strategies.' (014, Funder)

There might also be difficulties in interpreting the outcomes of nested studies, so including a qualitative element would be helpful:

'[You] would have to have a qualitative arm running in parallel with it; presumably that's what you encourage as well. So it would inform why certain recruitment strategies are working or not working. Because sometimes it's something bizarre that makes them work - not what you think is actually working.' (019 PI/Funder)

### How to nest recruitment interventions

Respondents suggested approaches to the design and implementation of nested recruitment interventions, highlighting timing, compatibility, confidence, communication, planning and resources. Notably, three of the funders emphasized that they viewed the piloting of recruitment methods as feasibility work which should normally be done before a trial began. Thus, although alternative recruitment methods could be evaluated, it would be important that they had been shown to be feasible. This could act as a major obstacle to the acceptance of nested recruitment interventions.

'I suppose what I am saying is I would be looking for evidence of feasibility; you know, I would have thought it should be demonstrated that a triallist can recruit by the time they come to a large grant.' (014, Funder)

#### Timing and resources

When asked whether nested recruitment studies should be introduced at the inception of the host trial, or as a 'rescue' if the trial encountered difficulties, most opted to include them from the outset, although a minority preferred to establish a recruitment baseline first.

'It's better to do it at the beginning when everyone is signed up to it, that's no question. I think that would be an advantage.' (012 PI)

'Bringing it in as an amendment in the middle would create trouble .... Because of the risks and difficulties and stuff that need to be addressed. Far better to do it as a package before you start.' (008, Ethics)

'I think it [establishing a baseline first] might have some advantages statistically, because you could do a controlled before and after analysis, so you could look to see whether giving extra education to clinicians leads to an increase in their recruitment.' (014, Funder)

It was seen as essential to plan and anticipate the resources needed for the added work that the nested study might entail.

'I suppose adequate resourcing in advance is the key, and being realistic about time frames for getting such a thing set up. .... Planning always takes longer than you expect, so I think, yes, enough anticipation of the time requirements and the resources needed is probably the key.' (016, TM)

#### Compatibility and communication

To enable integration there needed to be compatibility between the main study and the nested one. Respondents felt that early consultation with stakeholders would facilitate this.

'I think for it to work, you have to know that it's the right design for what you're doing in the context you are in, know it's well resourced enough to make it possible and not to jeopardize the main study. ... It's a very interesting idea if the proposal is one that's plausible and fits with the structure of the study ...' (003, PI)

'From the funder's perspective or the Programme Grant's perspective, the programmes need to be coherent, they need to fit together.... The whole needs to be based on the sum of the individual parts, and so the embedding of a nested study needs to be justified and in an ideal world it should be done at a pre-trial level.' (014, Funder)

Good communication and liaison between research teams was also considered important to build up trust and good working relationships.

'...so long as everybody understood the game plan and understood why it was happening...' (004, TM)

'If you're running your trial then you tend to be very involved with it and it tends to be your baby to a degree. You have other people coming in... you have to have a great level of trust. ... even if it's an independent project within a trial, there still has to be excellent liaison.' (019, PI/Funder)

Several of the principal investigators also commented that they would like recognition for having participated. One pointed out that if hosting recruitment research was to be a genuine collaboration, then the best incentive might be to be able to contribute to authorship:

'Oh, co-author on a Lancet paper and - done!' (020, PI)

### Reactions to potential recruitment interventions

During the interview, specific examples of potential recruitment interventions were presented to interviewees. Not all interviewees commented on each, but the key points made are summarized in table 3. A common theme was that hosts needed to have confidence in the intervention proposed.

**Table 3:** Summary of responses to proposals for nested recruitment studies

*Type of intervention*	*Perceived advantages*	*Perceived disadvantages*	*Points to consider in implementation*
Financial incentives to patients and professionals	Worth trying, extra resource; straightforward; it is justified to pay people for their time	May create ethical dilemmas, difficult to set right payment level; managing preferences may pose problems	May be more acceptable for professionals than patients; consult widely to set levels; avoiding coercion; avoiding drop-outs due to preferences?

Attachment of additional, dedicated research nurses for sessions in participating centres	Dedicated extra resource; logical; gives continuity within the research; creates ownership; stimulate interest on site	May impact on continuity of care; may cause logistical problems; more relationships to manage	Local input to staff selection; consider continuity of care; integration in practice; contractual issues

Use of DVD of previous trial participants discussing their experiences of trial participation	Worth trying; good idea; visual media are attractive; could work for lots of trials	Lack of time; unwillingness to watch; content may not be believed; may over-simplify; technical challenges	Mode of delivery, content, run-time; whether study specific or generic; age group biases; Information equity

Mass media approaches to change attitudes to trials among patients	Very important; good idea; may work well in areas with high refusal rates; challenge notion of 'guinea pig'	Expensive, difficult to focus message on local area or topic; may not produce immediate impact	Cost difficulties, measuring impact; avoiding bias

Educational incentives to clinicians: e.g. seminar on trials and research methods	Others report this works; may bring lasting benefit; research understanding will motivate participation	Lack of time; lack of interest; burden; difficult to motivate clinicians	Motivating clinicians; clinician preferences; how learning occurs; training location

Training for clinicians in seeking consent for trials	Interesting idea; may lead to more positive explanations of research; reduce clinician fear	Few studies use clinicians to consent patients; lack of time and motivation; burden	Assess numbers of studies using clinicians to consent; motivating clinicians; training location; control arm

Option to refer patients to a dedicated research centre	Feasible; interesting; participants will get more information and attention; professional	Additional cost and burden of travel; data collection and co-ordination	Defraying travel costs; coordinating data

Support for investigators on project management, monitoring and contingency planning	Good idea, but should be in place anyway	Difficult to randomize if only used by those who want help	Designing to enable randomization

'If recruitment was working or you could see that [nested approach to] recruitment had been researched and thought about, was based on evidence and was going to be successful, I can't see it being a problem...... if your trial estimated they were going to be reasonably successful, introducing different methods within individual clusters would be quite acceptable.' (011, TM)

Specific recruitment interventions could be problematic. Nested trials of financial incentives provoked strong reactions, such as this comment from an ethics committee chair.

'The one that most rings alarm bells is .... financial incentives and I think we've already discussed that about the balance between reasonable payment for reimbursement of expenses and over and above that - and does that payment become excessive and seen as an inducement when it may not necessarily be in an individual's best interest to take part in the study, but it shifts the balance and their ability to make an informed consent.' (007, Ethics)

Although some commented that from their experience, some of the recruitment interventions were beneficial, and hence not really worth testing, others pointed out potential disadvantages. For example, attaching a nurse researcher was felt by some to be an obvious stimulus to recruitment.

'The dedicated research nurses, again that seems to me a feasible trial within a trial, although it would seem to me so obvious that having extra staff would make a difference that you couldn't really claim too much equipoise on it; or at least I couldn't. And I couldn't sell equipoise to the practitioners. ....so I think it may be self-evident, although I couldn't quote any evidence for that at all.' (003, PI)

In contrast to this enthusiasm, others raised concerns that having additional staff coming into a practice might pose logistic problems or undermine continuity of care. Similar concerns were raised about whether other potentially beneficial recruitment interventions might increase the burden on potential participants or clinical collaborators. These are reported in Table 3 and illustrate the degree of uncertainty about the approaches suggested.

## Discussion

The present situation, where nested studies of recruitment methods are conducted on the initiative of individual investigators, means that there is no systematic approach to the choice of interventions. This in turn leads to concerns about the generalizability of findings beyond the individual trial and a failure to build knowledge about what works best over time. A more ambitious approach to improving the evidence base is through development of a portfolio of relevant recruitment interventions (based on theory and empirical evidence) which could be offered to investigators for inclusion within an individual trial, or across multiple trials, using the 'nested' methodology. If participation in nested studies was incentivized (using methods similar to those used to encourage greater public and patient participation in research), it is possible that a systematic database could be built of the effectiveness of different recruitment strategies, the variability in their effects, and the sorts of characteristics (of populations, interventions, or contexts) that moderate their effects. As noted in the introduction, there is an argument that the complexity of decision making about trial participation is such that the impact of any recruitment intervention will be influenced significantly by contextual factors, such as preferences for the interventions under test [21], other incentives in operation [22] and logistical barriers such as travel and cost [5]. By nesting recruitment interventions in a number of trials simultaneously, it would be possible to explore this hypothesis empirically, and determine whether the proposed approach to improving recruitment is likely to be fruitful.

What do the current data say about the successful implementation of such a model? At a very broad level, there was agreement that nested studies were a positive idea. This is expected, given the ubiquity of recruitment problems, and the expectation that trial investigators, funders and ethics committees would be positive about research in general. However, it was clear that given even limited time to consider the idea and some concrete examples, it was easy for respondents to identify a number of problems, and we concentrated our analysis on these challenges.

Representatives from funders gave the impression that they normally expected recruitment procedures to have been piloted and problems identified before embarking on the main study. Clearly, this is an explicit part of the MRC framework. However such pilots can be victim to the contextual issue raised above: where pilots are run in atypical contexts (e.g. enthusiastic practices who are early adopters of an intervention), the experience and the lessons derived may not reflect the response in a wider roll out. Nevertheless, the idea that nesting recruitment interventions might clash with the perception that the trial is viable is clearly a significant barrier.

Although some points related to scientific issues about design, the fundamental concerns related to the viability of the host trial in logistical and administrative terms when the nested trial was added. Participating in a nested recruitment study has implications for the time, attention and enthusiasm of researchers, and it appears that investigators will be making cost benefit assessments when deciding whether to participate. The difficulty is that, as for participants in most clinical trials, the benefits of nested recruitment studies accrue to the wider scientific community in the future, whereas the costs fall more immediately on the research team. This raises the issue of the importance of specific incentives to increase the perceived benefits of nested trials to their hosts. Clearly, many recruitment interventions could involve additional resources (e.g. nurse time, marketing advice, creation of resources etc) which could function as incentives, even if no direct financial incentive was in place. However, it is also clear that nested trials are likely to require additional resources for both the core research team running the nested studies and the host studies on which they are run.

A major theme related to issues of control. As noted previously, many published nested studies have been designed on the initiative of individual investigators, and one of the reasons for this is that in such cases they have control over both host and nested study. Nested studies conducted as part of a wider research initiative require that aspects of the design, analysis and write up may be taken 'outside' the research team (even if there is preliminary discussion and negotiation prior to adoption of the nested study). There was a strong suggestion that investigators would want to choose which interventions to test, and warnings that there could be a clash between the aims of the host and nested study. For example, if there was clear evidence from early data that one recruitment method was superior, it might be difficult to avoid the temptation to use it with all patients and sites, especially if overall recruitment is low. Equally, if anecdotal data suggested that an intervention was alienating collaborators, it might be difficult to continue with the nested study. Researchers, collaborators and patients within the host study are active agents, and there were suggestions that 'resentful demoralisation' [23] might occur in studies or sites which did not receive a favoured intervention like a financial incentive.

## Limitations of the study

It could be argued that seeking to randomize patients, professionals or trials to different recruitment interventions ignores the complexity of the recruitment process and attempts to apply a simplistic model to a complex area where context may be critical. However, the Medical Research Council framework that informed this study would emphasize that the methodology of nesting trials must be rooted in relevant theory, if the interventions to be nested are likely to be effective.

One major limitation of the study relates to the omission of patients. As noted by several respondents, nested studies may impact on patients, with the potential for confusion for patients if consent procedures were made more complex. It could be argued that in many cases patients will not need to know if they are part of a nested recruitment study. Indeed, seeking consent to participate in a trial of recruitment methods might impact on recruitment itself because simply telling a patient that they were being randomized to a recruitment incentive might influence their behaviour. Likewise clinical collaborators who are involved in recruitment might react in complex ways to being told that they were being randomized to different recruitment approaches. While we might have learnt more by including this group within our sample, we opted not to because our main focus was on the people directly concerned with designing and managing trials.

There were a variety of additional methodological limitations. Only a limited range of hypothetical scenarios were included, so there may be issues specific to certain types of trials that were not discussed. Although respondents were given information before the interview, not all had this to hand and they needed to respond quickly to issues that were raised during discussion. It is possible that other issues may have arisen after further consideration, or that their attitudes may have changed on subsequent reflection. It should be noted that some of the principal investigators and trials managers may not have recent experience of direct patient and professional recruitment. It is possible that the study may have benefited from the use of focus groups, although the logistics and cost of setting up such groups with dispersed respondents would have been prohibitive. The respondents from the funding bodies could only provide their personal perceptions and their views cannot be considered to reflect the policy of their employing organizations, although at least one respondent did circulate the interview schedule to a wider group of colleagues for comment. Although we explored differences between the four groups of respondents, these mainly followed their expected interests as researchers, funders and ethical guardians of patients' interests. We therefore concentrated on the issues common across groups in the analysis presented in this paper.

## Conclusions

Recruitment difficulties are widespread, and even though the creation of research networks has provided an infrastructure to address this, delays are still common and there are concerns that the small proportion of practices and patients participating in trials limits the generalizability of research findings. The current study has demonstrated some cautious support for the idea of nested studies of recruitment methods, and has provided insights into the barriers to their implementation. Key issues for consideration include planning at the earliest possible stage; working with principal investigators to ensure compatibility with the host study; and making sure that communication and relationships are effective. As with any study, the nested recruitment interventions must be rigorously developed to ensure findings are robust and generalisable. Principal investigators may have preferences about the timing of nested studies, but whether they begin at the start or after a baseline is established for the main study, clear procedures should be put in place and above all adequately resourced.

## Competing interests

The authors declare that they have no competing interests.

## Authors' contributions

All authors participated in the design of the study. EW recruited participants and carried out the interviews. EW, PB, JG, JM and DC analysed the interviews. The manuscript was drafted by EW, JG and PB. All authors read, commented on and approved the final manuscript.

## Pre-publication history

The pre-publication history for this paper can be accessed here:

http://www.biomedcentral.com/1471-2288/10/38/prepub
